# Associations Between Leisure-Time Physical Activity, Diet Type/Kind of Diet, and Body Weight Status Among Austrian Secondary School Pupils

**DOI:** 10.3390/nu18213614

**Published:** 2026-10-30

**Authors:** Clemens Drenowatz, Felix Polyudov, Werner Kirschner, Gerold Wirnitzer, Manuel Schätzer, Gerhard Ruedl, Katharina C. Wirnitzer

**Affiliations:** 1 Division of Sport, Physical Activity and Health, University of Education Upper Austria, 4020 Linz, Austria; 2 Department of Primary Education, University College of Teacher Education Tyrol, 6020 Innsbruck, Austria; felix.polyudov@gmail.com; 3 Department of Sport Science, Leopold-Franzens University of Innsbruck, 6020 Innsbruck, Austria; werner.kirschner@uibk.ac.at (W.K.); gerhard.ruedl@uibk.ac.at (G.R.); katharina.wirnitzer@uibk.ac.at (K.C.W.); 4 RockITLime, 6135 Stans, Austria; gerold@wirnitzer.at; 5 SIPCAN—Special Institute for Preventive Cardiology and Nutrition, 5061 Elsbethen, Austria; m.schaetzer@sipcan.at; 6 Department of Secondary Education, University College of Teacher Education Tyrol, 6020 Innsbruck, Austria; 7 Working Group Prevention, Integrative Medicine and Health Promotion, Department of Pediatric Oncology and Hematology, Otto-Heubner Centre for Paediatric and Adolescent Medicine (OHC), Charité—University of Medicine Berlin, 13353 Berlin, Germany; 8 Charité Competence Center for Traditional and Integrative Medicine (CCCTIM), Charité—Universitätsmedizin Berlin, Augustenburger Platz 1, 13353 Berlin, Germany

**Keywords:** leisure-time exercise, sports participation, plant-predominant diet, vegetarian, vegan, overweight, obesity, adolescents, school health promotion

## Abstract

**Background/Objectives:** Overweight and obesity present major public health challenges during adolescence. This study aimed to examine the independent associations of leisure-time physical activity (exercise frequency and club sports participation) and kinds of diet with body mass index (BMI) percentiles and weight status among Austrian secondary school pupils. **Methods:** Data were drawn from the nationwide cross-sectional *From Science 2 School* study. A total of 8799 pupils aged 9–22 years (63.1% female) completed an online survey capturing sociodemographic factors, exercise frequency, club sports participation, and kind of diet (omnivorous, vegetarian, vegan). Self-reported height and weight were used to calculate age- and sex-specific BMI percentiles. Data were analyzed using ANOVA with Bonferroni adjustment for post hoc tests as well as multivariable logistic regression analyses. **Results:** Overall, 11.8% of the participants were classified as overweight or obese and 82% reported regular leisure-time exercise. Engaging in leisure-time exercise on ≥4 days per week was associated with a 48% lower likelihood of overweight/obesity, with the strongest protective effect observed among 10–12-year-olds. Additionally, a vegetarian diet was associated with lower BMI percentiles (*p* < 0.01) and a 41% reduced risk of overweight/obesity, with effects being particularly prominent in girls. **Conclusions:** High-frequency leisure-time exercise and plant-based diet types are independently associated with more favorable body weight outcomes in adolescents. Integrated school-based prevention approaches addressing both physical activity and plant-centered nutrition may offer optimal synergies for obesity prevention.

## 1. Introduction

Overweight and obesity are major risk factors for a wide range of noncommunicable diseases and are associated with approximately 41 million deaths per year, which represents 74% of global mortality [1]. With the prevalence of overweight and obesity doubling from 1990 and 2022, the WHO refers to this development as a global crisis [2]. Among children and adolescents between 5 and 19 years of age, the global prevalence of overweight and obesity increased from 8% in 1990 to 20% in 2022, which accounts for more than 160 million children and adolescents [3]. There is, however, a large variability across countries with prevalence rates up to 40% in high-income countries. In Austria, the most recent national survey (2021–2022) showed self-reported overweight and obesity rates of 17% and 25%, among girls and boys, respectively [4].

Even though excess body weight ultimately reflects an imbalance between energy expenditure and energy intake, there is a complex interaction of biological and environmental factors that contribute to the regulation of body weight [3]. Physical activity (PA) and diet, however, are key behavioral components in weight management [5,6]. PA refers to any movement produced by skeletal muscles and is commonly classified into light, moderate, and vigorous intensity, corresponding to 40–63% of maximum heart rate (HR_max_), 64–76% HR_max_, and 77–95% HR_max_, respectively [7]. Leisure-time PA includes sport and exercise, subdomains of PA, which are often goal-oriented (e.g., enjoyment, health, athletic performance). Sports are an additional subcomponent of PA, which are frequently institutionally organized (e.g., within a club) and may include competition [8,9].

Available data on the PA levels of children and adolescents, however, show a high prevalence of insufficient PA across all age groups [10,11,12,13]. Globally, only 15% of girls and 25% of boys aged 11–15 years meet current recommendations of at least 60 min of moderate-to-vigorous PA per day [9,12]. In Austrian youth between 5 and 17 years of age only 10% and 20% of girls and boys, respectively, achieved 60 min of daily moderate-to-vigorous PA [14]. Similarly, dietary habits among adolescents deviate substantially from recommendations. International data indicate that only 38% of adolescents consume fruits and vegetables daily [12]. In Austria, only 41–62% of girls and 29–55% of boys consume fruit at least once daily, while daily vegetable consumption ranged 43–49% among girls and 29–42% among boys [14]. Large scale studies, however, primarily assess dietary behavior through individual food consumption (e.g., fruit and vegetable intake) [15,16,17] rather than considering kind of diet (omnivorous, vegetarian, and vegan). Even though the benefits of regular PA and dietary habits for weight management have been well documented [6,18], there remains limited data on the associations of diet types and leisure-time exercise as well as club sports participation with BMI in Austrian adolescents. By exploring these associations, the goal of the present study was to improve the understanding of lifestyle-related determinants of overweight and obesity in the Austrian school setting and to underscore the need for targeted school-based prevention strategies.

## 2. Materials and Methods

The *From Science 2 School* study was designed as a multidisciplinary cross-sectional study using a multistage cluster sampling strategy. The study was supported by the Austrian Federal Ministry of Education, Science and Research (BMBWF, Department I/7: School and University Sport) and approved by all nine Austrian School Boards. Details of the study protocol have been published previously [19] and are available on the official project website (https://www.science2.school/en/ accessed on 27 September 2026).

Briefly, all lower and upper secondary school pupils in Austria were invited to participate in the study anonymously and voluntarily. The survey was made available nationwide using LimeSurvey (LimeSurvey GmbH, Hamburg, Germany; version 3.17.16) via an encrypted online platform for anonymous participation and could be completed using smartphones, computers, or tablets. Invitations were distributed by email to all 2688 secondary schools in Austria. Although data collection spanned from 28 October 2019 to 10 July 2020, 98% of participants completed the survey prior to 10 March 2020, preceding the implementation of any COVID-19-related policy implementation and associated restrictions on school and club sports in Austria. Prior to starting the online questionnaire, information on the nature of the study was provided and participants were informed that they could withdraw from the study at any time. Teachers were also informed about the study objectives and procedures and pupils completed the questionnaire either during class or at home under the supervision of a legal guardian. The completion and submission of the questionnaire implied consent.

The questionnaire collected sociodemographic information (e.g., school level, sex, age), anthropometric data (body weight and height), information on participation in leisure-time exercise and club sports (including the number of exercise days per week), and dietary characteristics. Dietary subgroups were classified as omnivorous (mixed diet including animal products such as meat and dairy products), vegetarian (excluding meat and fish but including dairy products and eggs), or vegan (consisting exclusively of plant foods) [20,21]. These labels were treated as mutually exclusive analytic categories as recorded in the questionnaire. Conceptually, however, vegan diets are a subset of vegetarian diets, and final classification was determined based on answers to control questions on the consumption of specific foods. BMI was calculated (kg/m^2^) using self-reported body weight and height and was converted to age- and sex-specific percentiles based on German reference values [22].

### Statistical Analysis

Data completeness was assured by requiring response fields for core demographic, behavioral, and anthropometric items in the online interface. Descriptive statistics are presented as prevalence for categorical variables and means with standard deviation for continuous variables. Differences in diet type, leisure-time exercise, and club sports by sex and age group were analyzed using chi-squared tests. Associations of diet type, leisure-time exercise, and club sports participation with BMI percentile were examined using analysis of variance (ANOVA) with Bonferroni correction for post hoc analyses. Cohen’s d and eta-squared values are used as indicators for effect sizes. Given the large sample size (N = 8799), the central limit theorem ensures robustness against minor departures from normality. Nevertheless, normality of residuals was confirmed with values for skewness and kurtosis of less than 0.11 and 1.25, respectively, across group comparisons. Furthermore, homogeneity of variance was calculated as the ratio of the largest group variance to the smallest group variance for the respective group comparisons due to the over-sensitivity of Levene’s test in large samples. Values ≤ 1.15 fell well below the standard 3:1 guideline where ANOVA robustness would be compromised. In addition, logistic regression analysis was used to test whether dietary habits and the weekly number of exercise days were associated with overweight and obesity. To evaluate the predictive validity of the multivariable logistic regression model goodness of fit and explained variance were examined using Nagelkerke’s R^2^. Multicollinearity among independent variables was assessed using variance inflation factors (VIFs) and tolerance statistics, with all VIF values remaining <1.05 (tolerance > 0.95), indicating an absence of significant collinearity. All analyses were conducted for the total sample and stratified by sex and age group. A second analysis also included living environment (urban vs. rural) as a covariate. All statistical analyses were performed using IBM SPSS Statistics version 29.0 (IBM Corp., Armonk, NY, USA) with statistical significance set at *p* < 0.01.

## 3. Results

A total of 8845 participants completed the online questionnaire. Forty-six pupils, however, were excluded due to implausible anthropometric values (height < 110 cm, body weight < 20 kg, BMI < 10 kg/m^2^, or BMI > 50 kg/m^2^). The final analytical sample therefore consisted of 8799 participants (63.1% female and 36.9% male) aged 9–22 years. A majority of the participants (68.3%) lived in a rural environment. Table 1 summarizes the anthropometric characteristics of the final sample and the sex-specific subsamples. Although girls were, on average, older than boys (*p* < 0.01), boys were taller and had a higher body weight (*p* < 0.01). Boys also displayed higher BMI percentiles and a higher prevalence of overweight and obesity than girls (*p* < 0.01). Overall, approximately three quarters of the participants were classified as having normal weight according to BMI percentile, while 11.8% were classified as overweight or obese.

### 3.1. Leisure-Time Physical Activity and Diet

Across the total sample, 82.4% (n = 7253) reported participating in leisure-time exercise, with 35.8% (n = 3154) reporting at least four days per week of exercise engagement. Furthermore, 35.0% (n = 3083) participated in club sports. Boys reported higher participation in both leisure-time exercise and club sports than girls (*p* < 0.01). They also engaged in leisure-time exercise on at least four days per week more frequently than girls (46.6% vs. 29.5%, *p* < 0.01).

Participation in leisure-time exercise and club sports declined significantly with increasing age (*p* < 0.01). Similarly, engagement in leisure-time exercise on at least 4 days per week declined significantly with age from 50.8% among participants aged 10–12 years to 27.2% among participants aged 17 years or older (*p* < 0.01; Table 2).

An omnivorous diet was the predominant diet while plant-based kinds of diet were reported by less than 10%. Vegetarian and vegan diets were reported significantly more often among girls compared to boys (*p* < 0.01). Across age groups, the prevalence of an omnivorous diet decreased, whereas the prevalence of a vegetarian diet increased (*p* < 0.01; Table 2).

### 3.2. Association of Leisure-Time Physical Activity, Diet Type and Body Weight

**Figure 1:**
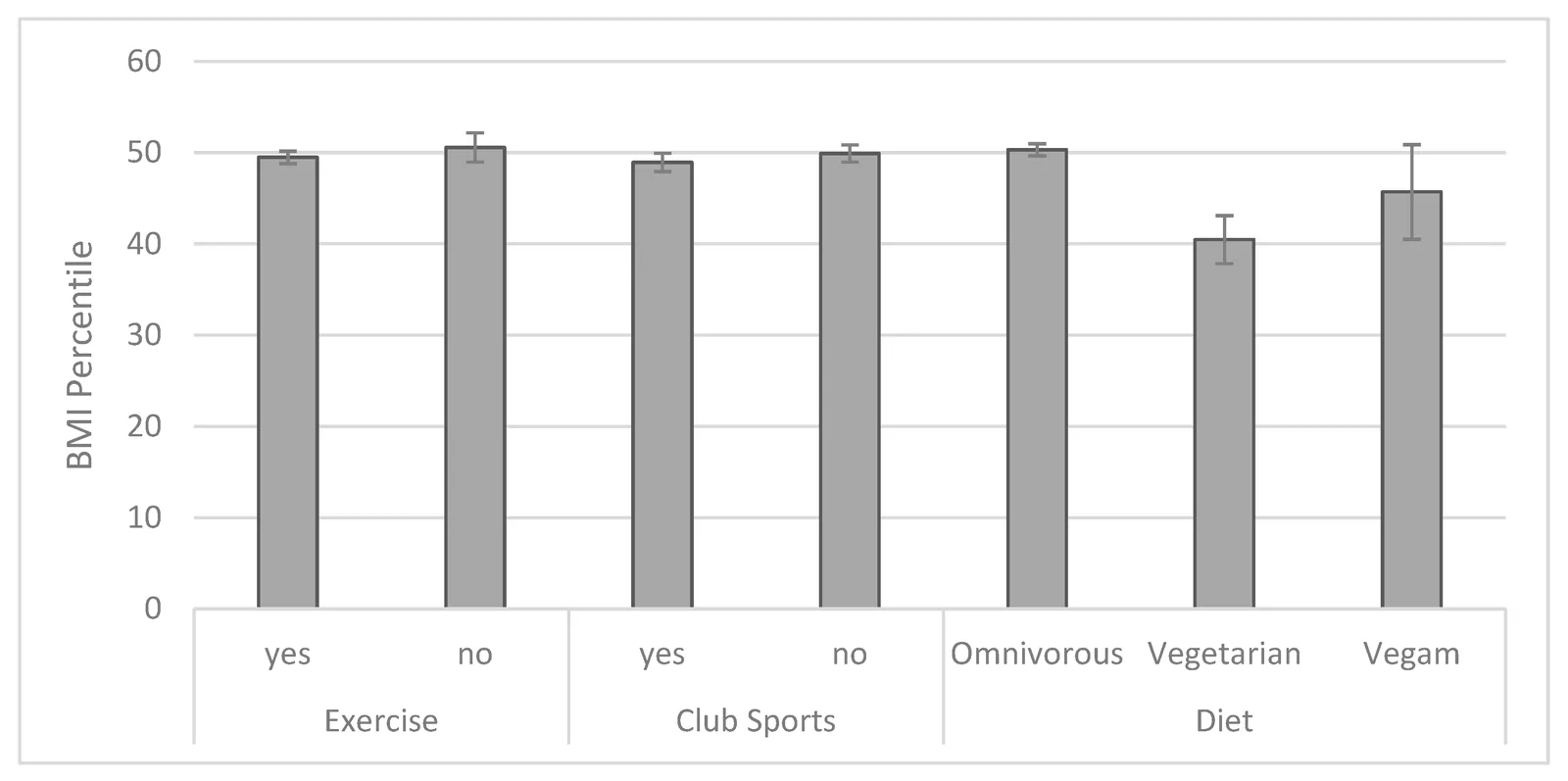
BMI percentile by leisure-time exercise and club sports participation as well as diet. Values are means with 95% confidence intervals.

In the total sample, no significant interaction effect of leisure-time exercise or club sports participation and kind of diet with BMI percentile was found. Diet type, however, was significantly associated with BMI percentile while there was no difference in BMI percentile by participation in leisure-time exercise and club sports (Figure 1). Specifically, participants following a vegetarian diet displayed lower BMI percentiles than those following an omnivorous diet (*p* < 0.01). Following a vegan diet, on the other hand, was not associated with a significantly different BMI percentile from those with an omnivorous or vegetarian diet. These results remained essentially the same after including living environment (urban vs. rural) as covariate in the analyses.

Subgroup analyses did not show significant associations between leisure-time exercise and BMI percentiles either, except for participants between 10 and 12 years of age. Club sports participation, on the other hand, was associated with lower BMI percentiles among boys and those younger than 15 years (Table 3), while BMI percentiles did not differ significantly across diet types. A vegetarian diet, on the other hand, was associated with lower BMI percentiles among girls and participants above 15 years of age (Table 4). As for the total sample, the inclusion of living environment as covariate did not affect the reported outcomes.

Logistic regression analyses further showed a reduced likelihood of overweight and obesity with increasing frequency of leisure-time exercise even though the overall model explained only a small proportion of the variance in weight status (Nagelkerke’s R^2^ between 0.013 and 0.045). Given the complex interaction of genetics, behaviors and the environment regarding body weight, the primary aim of this study, however, was to examine the specific adjusted associations of leisure-time exercise and diet with the risk for overweight/obesity rather than predicting BMI or weight status based on behavioral choices. Compared to no regular leisure-time exercise, engagement on 1–3 days per week was associated with a 26% risk reduction for overweight and obesity, and engagement on 4–7 days per week was associated with a 48% risk reduction (*p* < 0.01; Table 5). A vegetarian diet was also associated with a significant reduction in the likelihood of overweight and obesity compared to an omnivorous diet (*p* < 0.01). Even though a similar risk reduction was observed for a vegan diet, the result was not significant due to the wide confidence interval.

Sex-specific analyses showed that a vegetarian diet was associated with a lower likelihood of overweight and obesity among girls (*p* < 0.01), whereas no significant association between diet type and overweight or obesity was observed among boys or within the age-group analyses (Table 5). In contrast, the inverse association between leisure-time exercise frequency and overweight and obesity was observed consistently among boys and girls, particularly with leisure-time engagement on at least 4 days per week. A higher frequency of leisure-time exercise engagement was also associated with a lower risk for overweight across age groups, except for those between 13 and 14 years of age, while dietary pattern was not significantly associated with weight status across age groups. As reported for BMI percentiles, results of the logistic regression analyses remained essentially the same after adjusting for living environment.

## 4. Discussion

The results of the present study show higher leisure-time exercise among boys compared to girls. Furthermore, participation rates decreased with increasing age, while a plant-based diet became more common with increasing age, even though an omnivorous diet was the dominant diet type. Girls were more likely to report a plant-based diet. A vegetarian diet was also associated with a lower body weight, particularly among girls and older participants. Girls with a vegetarian diet further displayed a lower prevalence of overweight and obesity compared to those with an omnivorous diet. Among boys and younger participants, on the other hand, club sports participation was associated with a lower body weight. Furthermore, a higher frequency of leisure-time exercise participation was associated with a lower risk for overweight and obesity, while the association between overweight and obesity with diet type was not significant in the age group-specific analyses.

The prevalence of overweight and obesity in the present study (11.8%, including 4.7% with obesity), however, was lower than that reported in global and national surveillance studies [4,15,16,23,24,25]. According to the WHO, the global prevalence of overweight (including obesity) among 5- to 19-year-olds reached 20% in 2022, while the prevalence of obesity reached 8% [3]. In Europe, the latest COSI survey reported that 25% of children aged 7–9 years were affected by overweight, including 11% with obesity [23], while national German data using anthropometric measurements, rather than self-reported data, showed prevalence of 15% and 6% for overweight and obesity, respectively, among 3- to 17-year-olds [26]. Austrian data based on self-reported height and weight also indicates a prevalence of 17% overweight/obesity among adolescents, with higher prevalence among boys [27]. Results on exercise behavior in the present study, on the other hand, correspond to physical activity patterns that have been observed previously. International as well as national data have shown higher physical activity levels and more frequent engagement in club sports among boys along with a decline in physical activity with increasing age [11,13,14].

Regular exercise, particularly engagement in leisure-time exercise on at least four days per week, was associated with a lower likelihood for overweight and obesity, with a stronger association among younger participants. Nevertheless, the modest pseudo-R^2^ values highlight that the regulation of body weight in adolescents is multifactorial, involving complex genetic, metabolic, socioeconomic, and environmental determinants beyond exercise and diet. Previous studies, including those using objective PA and anthropometry data, however, also demonstrated a protective effect of PA against overweight and obesity [28,29,30,31]. In contrast, associations of leisure-time exercise and club sports participation with BMI percentiles were limited, which might suggest that leisure-time exercise and sports protect against an extreme manifestation of excess body weight rather than lowering body weight in general. Furthermore, a minimum amount of engagement may be required in order to induce benefits for weight management as subgroups with higher leisure-time exercise engagement showed significant associations with BMI. It should also be considered that BMI does not distinguish between fat mass and lean body mass and more active adolescents may have a higher lean body mass, which could result in similar BMI percentile values despite lower fat mass [32,33]. A plant-based diet, on the other hand, was associated with a lower body weight as well as a lower prevalence of overweight and obesity. The stronger association among girls and older participants may be attributed to a higher prevalence of plant-based diets in these subgroups. Previous studies also showed beneficial associations of vegetarian and vegan diets with self-reported body weight [34,35,36,37,38]. Furthermore, plant-based diets have been associated with a more favorable body composition [33,35,39,40] and improved cardiometabolic health outcomes [41].

Even though overweight and obesity rates were low in the present study, it should be considered that childhood and adolescence represent critical periods for developing health-related knowledge, competencies, and behaviors, which affect future health. Adolescence, however, is also characterized by an increased risk for developing and consolidating unhealthy lifestyle habits [42], which often persist into adulthood [43]. As insufficient PA and unhealthy dietary habits contribute to the development of overweight and obesity, and are major risk factors for noncommunicable diseases such as hypertension, type 2 diabetes mellitus, and cardiovascular disease [1], additional efforts are needed to promote an active and healthy lifestyle. In Austria alone, the productivity losses attributable to insufficient PA were estimated at approximately €248 million in 2017 [44]. Accordingly, improving young people’s health is not only an individual objective but also an important foundation for population health.

School-based health promotion can help to empower pupils to take greater responsibility for their own health, and the Austrian curriculum specifically addresses health literacy as an overarching educational objective and a cross-curricular teaching principle [45]. Schools provide an ideal setting for health promotion as they reach children and adolescents regardless of socioeconomic background. Through the nine years of compulsory education in Austria (8–13 years in other EU countries), youth also spend a substantial proportion of their time in school with school and school-related activities, accounting for approximately 40% of pupils’ waking hours on regular school days [46]. Physical education plays a central role in the promotion of an active lifestyle and particularly, high-quality physical education can improve motor competence and thereby facilitate participation in leisure-time exercise [47,48,49]. Furthermore, nutritional aspects need to be addressed, as a combined approach that integrates PA promotion with nutrition education provides greater health benefits than interventions focusing on a single lifestyle domain [5,50]. A recent systematic review and meta-analysis of adolescents aged 10–19 years emphasized this integral approach [31] and international public health recommendations emphasize a combination of PA and healthy dietary choices [51,52], which is increasingly recognized as HEAL (healthy eating and active living) [53].

From a broader translational perspective, these findings hold substantial implications for healthcare policy, clinical practice, and future research. At the healthcare policy and school administration level, the observed steep decline in leisure-time exercise across adolescence underscores an urgent need for structural interventions. Policies should prioritize bolstering high-quality, inclusive physical education and expanding subsidized community or school-affiliated sports programs to reverse age-related activity drop-offs, particularly among female adolescents [54]. In clinical practice, pediatricians, family physicians, and school nurses should move beyond single-behavior counseling by adopting integrated healthy eating and active living (HEAL) frameworks [53]. Because plant-based diets were associated with more favorable weight outcomes, especially among girls, clinical guidance should support safe, well-planned plant-based dietary options rather than focusing exclusively on physical activity parameters [55].

Several limitations of this study, however, should be considered when interpreting the findings. All data were collected using a standardized self-report questionnaire and are therefore susceptible to reporting bias. Particularly height and body weight may have been misreported due to social desirability bias, with adolescents tending to underestimate body weight and overestimate height, which results in an underestimation of BMI-based estimates of overweight and obesity prevalence [56]. This phenomenon may have contributed to the low prevalence of overweight and obesity in the present study. Additionally, voluntary participation may have contributed to the (on average) healthier weight status across the sample as pupils with a greater interest in PA and nutrition may have been more likely to participate. A higher health consciousness, however, could also have contributed to higher data quality, as motivated participants potentially provide more accurate and complete information. The uneven distribution of exercise participation may further have contributed to the lack of significant findings regarding the participation in exercise in general as approximately 80% of participants reported regular leisure-time exercise participation while only 20% were considered non-exercisers. An additional limitation is the lack of information on intensity or duration of leisure-time exercise, which limits comparability with studies evaluating compliance with current PA recommendations. Even though the high prevalence of an omnivorous diet is to be expected, the vegan subgroup comprised only 1.6% of the sample, limiting statistical power. While the associations with BMI percentiles and weight status observed for the vegan diet were similar to those for the vegetarian diet, they did not reach statistical significance. The lack of statistical significance, therefore, is most likely a reflection of a Type II error due to insufficient statistical power and the wide confidence intervals in this small subgroup. Consequently, these findings should be interpreted with caution, and future research with larger, purposefully oversampled cohorts of vegan adolescents is necessary to verify these trends. Data collection also overlapped with the onset of the COVID-19 pandemic in early 2020. Nevertheless, 98% of the sample responded prior to the enforcement of any COVID-related policies, which started with travel restrictions. The present data, therefore, capture pre-pandemic behavioral patterns regarding leisure-time exercise, club sports, and dietary habits without the confounding influence of pandemic-related activity restrictions. Finally, the cross-sectional design of this study does not permit causal inferences. For example, a higher BMI may reduce participation in leisure-time exercise rather than being a precursor for low participation. Despite these limitations, the large nationwide sample (N = 8799; approximately 1.1% of the Austrian secondary school pupils) represents a major strength of the study. The nationwide recruitment, with invitations distributed to all 2688 Austrian secondary schools and participation available across all nine federal states, supports a broad coverage. Nevertheless, the reliance on voluntary participation rather than a probability sample with population weighting along with the sample’s sex distribution (63.1% female) and lower observed overweight/obesity prevalence than national surveillance estimates indicate that it should not be considered fully representative of the target population. The inclusion of plausibility checks and control questions throughout the questionnaire, however, improve data quality on dietary habits and leisure-time exercise. The present study therefore provides important empirical evidence on the independent associations of leisure-time exercise and kind of diet with body weight among Austrian youth.

## 5. Conclusions

Overall, the present findings support the growing body of evidence indicating that regular exercise participation and a plant-based diet are independently associated with body weight among adolescents. Given that overweight and obesity are among the most important preventable health risks during childhood and adolescence, these findings emphasize the importance of evidence-based prevention strategies. While frequent participation in leisure-time exercise was primarily associated with a lower likelihood of overweight and obesity, plant-based diet types were associated with a lower body weight in general, especially among girls and older adolescents, who are generally less active. Preventive strategies that encourage regular exercise and healthy dietary habits, therefore, have the potential to substantially contribute to a healthy body weight during adolescence, which affects future public health. Additional research that transitions from cross-sectional designs to large-scale prospective cohort studies and randomized controlled trials incorporating objective physical activity monitoring and precise body composition assessments, however, is needed to enhance the understanding of the association between emerging plant-predominant dietary patterns, habitual exercise frequency, and metabolic health, which will be vital to optimize targeted, gender-sensitive obesity prevention initiatives during the critical adolescent transition.

## Figures and Tables

**Table 1:** Anthropometric characteristics of the study population. Values are means ± standard deviation and prevalence for weight categories.

	TOTAL	Girls	Boys
Age (years) ^1^	15.1 ± 2.3	15.4 ± 2.2	14.7 ± 2.3
Height (cm) ^1^	166.6 ± 10.5	164.2 ± 7.9	170.6 ± 13.0
Weight (kg) ^1^	58.5 ± 14.3	56.2 ± 11.6	62.4 ± 17.3
BMI percentile ^1^	49.7 ± 30.4	45.3 ± 30.2	57.3 ± 29.2
* * *Normal Weight*	75.8% (n = 6667)	75.0% (n = 4161)	77.1% (n = 2506)
* * *Underweight*	12.4% (n = 1095)	15.4% (n = 855)	7.4% (n = 240)
* * *Overweight*	7.1% (n = 625)	5.9% (n = 325)	9.2% (n = 300)
* * *Obese*	4.7% (n = 412)	3.8% (n = 209)	6.2% (n = 203)

^1^ Significant sex differences (*p* < 0.01).

**Table 2:** Prevalence of participation in leisure-time exercise, club sports and diet.

	Leisure-Time Exercise and Club Sports			Diet		
	Leisure-Time Exercise	≥4 Days/Week	Club Sports	Omnivorous	Vegetarian	Vegan
**Total**	82.4% (n = 7253)	35.8% (n = 3154)	35.0% (n = 3083)	92.8% (n = 8164)	5.6% (n = 493)	1.6% (n = 142)
Girls	80.5% (n = 4466)	29.5% (n = 1639)	29.5% (1635)	90.3% (n = 5013)	7.7% (n = 429)	1.9% (n = 108)
Boys	85.8% (n = 2787)	46.6% (n = 1515)	44.6% (1448)	97.0% (n = 3151)	2.0% (n = 64)	1.0% (n = 34)
10–12 years	92.7% (n = 1149)	50.8% (n = 630)	47.8% (592)	94.1% (n = 1166)	2.8% (n = 35)	3.1% (n = 38)
13–14 years	86.9% (n = 1778)	43.2% (n = 885)	39.6% (811)	94.8% (n = 1941)	3.9% (n = 79)	1.3% (n = 27)
15–16 years	79.7% (n = 2435)	31.7% (n = 969)	33.4% (1022)	92.4% (n = 2821)	6.3% (n = 193)	1.3% (n = 40)
>17 years	76.9% (n = 1891)	27.2% (n = 670)	26.8% (658)	90.9% (n = 2236)	7.6% (n = 186)	1.5% (n = 37)

**Table 3:** BMI percentile by leisure-time exercise and club sports participation for the total sample and separately by sex and age. Values are means with standard deviation.

	Leisure-Time Exercise					Club Sports				
	Yes	No	*F*	*p*	*Cohen’s d*	Yes	No	*F*	*p*	*Cohen’s d*
**Total**	49.5 ± 30.0	50.6 ± 32.2	*1.607*	*0.205*	*0.036*	48.9 ± 28.8	49.9 ± 30.9	*1.880*	*0.170*	*0.033*
Girls	44.9 ± 29.9	46.7 ± 31.4	*3.071*	*0.080*	*0.126*	43.8 ± 28.5	45.5 ± 30.7	*3.369*	*0.067*	*0.057*
Boys	56.9 ± 28.7	59.7 ± 32.1	*3.745*	*0.053*	*0.196*	54.7 ± 28.0	59.2 ± 29.2	*17.181*	*<0.001*	*0.232*
10–12 years	49.8 ± 30.5	60.4 ± 30.9	*10.017*	*0.002*	*0.346*	47.4 ± 30.1	52.4 ± 30.8	*7.974*	*0.005*	*0.167*
13–14 years	53.2 ± 29.9	55.5 ± 30.6	*1.376*	*0.241*	*0.077*	50.7 ± 28.4	55.3 ± 30.9	*10.293*	*0.001*	*0.153*
15–16 years	50.8 ± 28.7	53.1 ± 31.2	*2.894*	*0.089*	*0.077*	50.5 ± 27.7	51.1 ± 29.4	*0.243*	*0.622*	*0.020*
>17 years	44.1 ± 30.7	44.0 ± 33.0	*0.007*	*0.932*	*0.004*	45.7 ± 29.4	43.2 ± 31.3	*2.828*	*0.093*	*0.081*

**Table 4:** BMI percentile by diet pattern for the total sample and separately by sex and age. Values are means with standard deviation.

	Omnivorous	Vegetarian	Vegan	*F*	*p*	*Eta-Squared*
**Total ^1^**	50.3 ± 30.2	40.5 ± 29.7	45.7 ± 31.3	*25.745*	*<0.001*	*0.006*
Girls ^1^	45.9 ± 30.2	38.4 ± 28.9	41.5 ± 30.8	*13.210*	*<0.001*	*0.005*
Boys	57.3 ± 29.1	54.6 ± 31.0	59.1 ± 29.4	*0.339*	*0.713*	*0.000*
10–12 years	51.2 ± 30.6	38.0 ± 32.0	42.7 ± 30.4	*4.491*	*0.011*	*0.007*
13–14 years	53.6 ± 30.0	48.3 ± 28.8	62.2 ± 30.3	*2.320*	*0.099*	*0.002*
15–16 years ^1^	52.0 ± 29.2	41.3 ± 28.5	50.1 ± 28.7	*12.081*	*<0.001*	*0.008*
>17 years ^1^	44.9 ± 31.2	36.7 ± 30.3	32.0 ± 30.0	*8.736*	*<0.001*	*0.007*

^1^ Significant difference between omnivorous and vegetarian diet (*p* < 0.01).

**Table 5:** Association of leisure-time sport (active days/week; reference: no leisure-time exercise) and kind of diet (reference: omnivorous diet) with overweight and obesity. Values are odds ratios with 95% confidence intervals.

	Model Fit Nagelkerke’s R^2^	Exercise on 1–3 Days/Week	Exercise on 4–7 Days/Week	Vegetarian	Vegan
**Total** (N = 8799)	0.014	0.74 (0.63; 0.87) ^1^	0.52 (0.43; 0.62) ^1^	0.59 (0.42; 0.83) ^1^	0.57 (0.30; 1.09)
**Girls** (n = 5550)	0.014	0.79 (0.63; 0.98)	0.53 (0.41; 0.69) ^1^	0.57 (0.38; 0.86) ^1^	0.57 (0.25; 1.32)
**Boys** (n = 3249)	0.027	0.66 (0.51; 0.85) ^1^	0.40 (0.30; 0.52) ^1^	1.21 (0.64; 2.30)	0.69 (0.24; 1.97)
**10–12 years** (n = 1239)	0.045	0.41 (0.24; 0.70) ^1^	0.25 (0.14; 0.42) ^1^	0.90 (0.31; 2.59)	0.18 (0.03; 1.35)
**13–14 years** (n = 2047)	0.014	1.05 (0.72; 1.52)	0.63 (0.42; 0.93)	0.61 (0.28; 1.34)	0.80 (0.24; 2.67)
**15–16 years** (n = 3054)	0.017	0.63 (0.48; 0.82) ^1^	0.49 (0.36; 0.66) ^1^	0.58 (0.33; 1.00)	0.62 (0.19; 2.02)
**>17 years** (n = 2459)	0.013	0.74 (0.54; 1.00)	0.52 (0.36; 0.76) ^1^	0.61 (0.34; 1.12)	0.80 (0.24; 2.64)

^1^ Significant association with overweight and obesity at *p* < 0.01.

## Data Availability

The data presented in this study are not publicly available due to Austrian data protection and data security laws, with particular emphasis on the educational guidelines and regulations governing the conduct of scientific research involving children and adolescents (considered a vulnerable sub-population) as well as educational staff in the Austrian school system. Requests to access the data should be directed to the PI of the study (katharina.wirnitzer@ph-tirol.ac.at).
